# Efficient graphene saturable absorbers on D-shaped optical fiber for ultrashort pulse generation

**DOI:** 10.1038/srep20644

**Published:** 2016-02-09

**Authors:** J. D. Zapata, D. Steinberg, L. A. M. Saito, R. E. P. de Oliveira, A. M. Cárdenas, E. A. Thoroh de Souza

**Affiliations:** 1 MackGraphe - Graphene and Nanomaterials Research Center, Mackenzie Presbyterian University, São Paulo/SP, Brasil; 2 Universidad de Antioquia, Medellin, Colombia

## Abstract

We demonstrated a method to construct high efficiency saturable absorbers based on the evanescent light field interaction of CVD monolayer graphene deposited on side-polished D-shaped optical fiber. A set of samples was fabricated with two different core-graphene distances (0 and 1 μm), covered with graphene ranging between 10 and 25 mm length. The mode-locking was achieved and the best pulse duration was 256 fs, the shortest pulse reported in the literature with CVD monolayer graphene in EDFL. As result, we find a criterion between the polarization relative extinction ratio in the samples and the pulse duration, which relates the better mode-locking performance with the higher polarization extinction ratio of the samples. This criterion also provides a better understanding of the graphene distributed saturable absorbers and their reproducible performance as optoelectronic devices for optical applications.

The graphene is a two-dimensional carbon allotrope nanomaterial consisting of one atom thickness and that has important optical and electronic properties^1^,^2^. Due to its optical properties graphene is used for applications such as ultrashort pulse generation^2^,^3^,^4^,^5^,^6^,^7^,^8^,^9^,^10^,^11^,^12^,^13^,^14^,^15^,^16^,^17^,^18^, wavelength converter^19^,^20^, optical modulation^2^,^21^ and polarizing^22^. There are two different main mechanisms for propitiating the interaction between light and graphene in optical fiber based devices: 1) the transference of the graphene on the optical fiber face tip^3^,^4^,^5^,^6^ and 2) the transference of the graphene close to the fiber’s core along the propagation direction. In this second case, the interaction occurs through the evanescent light field^10^,^11^,^12^,^13^,^14^,^15^,^16^,^17^,^18^ in tapered segments of a fiber^23^ or in the polished surface of D-shaped optical fibers^10^,^11^,^12^,^13^,^14^,^15^,^16^,^17^,^18^ where graphene was transferred. Considering the graphene’s atomic thickness, the light-graphene length interaction can be highly enhanced by the geometry of evanescent field as light propagates parallel to the graphene.

Ultrashort pulses are suitable for numerous applications, which include telecommunications and the study of ultrafast processes^3^,^4^,^5^,^6^,^24^. The generation of ultrashort pulses in mode-locked EDFL using graphene as saturable absorber (SA) is a relatively affordable option^2^,^3^,^4^,^5^,^6^,^7^,^8^,^9^,^10^,^11^,^12^,^13^,^14^,^15^,^16^,^17^,^18^ and was first demonstrated by Hazan *et al.* using exfoliated graphene dispersed in a polymer at all-fiber connector tip configuration, which pulses as short as ~800 fs with 3.2 nm bandwidth^3^ were generated. Graphene fabricated through CVD process has also been considered for mode-locking EDFLs with the advantage of a better control on graphene’s production. Using multilayer CVD graphene also at all-fiber connector tip configuration, Bao *et al.* demonstrated the generation of 756 fs pulses with 5 nm spectrum bandwidth^4^. Posteriorly, it was obtained 1.23 ps pulses with CVD monolayer graphene saturable absorber^5^. Hereafter, pulses of 570 fs^6^ in short-length cavities as well as 415 fs^7^ in long-length cavities were demonstrated with multilayer CVD graphene samples. Pulse duration as short as 315 fs was obtained in EDFL using a CVD bilayer graphene samples^8^. The generation of ultrashort pulses using graphene deposited on D-shaped side-polished optical fiber is an alternative to improve the light-graphene interaction, in addition to provide the development of compact and robustness optical devices. It was demonstrated a saturable absorber by using a CVD monolayer graphene on the D-shaped optical fiber in an EDFL generating 668 fs pulses with 7.8 nm bandwidth^10^. Also, there are several reports using graphene D-shaped optical fiber saturable absorbers for ultrashort pulses generation^10^,^11^,^12^,^13^,^14^,^15^,^16^,^17^,^18^, the shortest pulse reported is 423 fs by using electric gate control on D-shaped optical fiber with bilayer graphene^18^, but none of them demonstrated a detailed mode-locking performance criterion of graphene saturable absorber.

In this work, we present an efficient and reproducible mode-locking performance criterion with seven D-shaped side-polished fiber samples based on CVD monolayer graphene saturable absorbers for EDFL, obtaining pulses duration of 256 fs, which is the shortest pulse reported in the literature with CVD graphene monolayer, for our knowledge. We carried out the characterization process in three steps after the fabrication of the sample by the transference of the graphene to the D-shaped optical fiber: the first step was to verify the quality of the monolayer graphene by Raman spectroscopy. Thereafter, the performance of the monolayer graphene sample as a polarizer was evaluated by the polarization relative extinction ratio between the modes of the light with polarization parallel (TE radiation) and orthogonal (TM radiation) to the graphene’s plane. Finally, we characterized the graphene saturable absorber mode-locking performance relating it to the polarization relative extinction ratio of each sample.

## Results

### Graphene quality characterization by Raman spectroscopy

**Figure 1:**
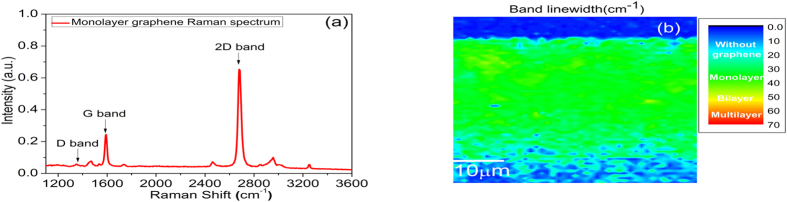
Raman spectrum. **(a)** Raman spectrum of the monolayer transferred graphene to D-shaped optical fiber and **(b)** 2D band linewidth mapping of a 46 μm × 37 μm surface area showing mostly monolayer graphene.

The samples were analyzed with a Raman Microscopy System (Alpha 300 R Confocal) using a 532 nm wavelength laser with an incident power of 2 mW. In Fig. 1(a), it is shown the Raman spectrum of the monolayer transferred graphene with the D (1350 cm^−1^), G (1592 cm^−1^) and 2D (2685 cm^−1^) characteristic bands of the graphene^25^. The graphene length and core-surface distance of this sample were 25 mm and 1 μm, respectively. Also, as shown in Fig. 1(b), a 2D band linewidth Raman mapping of a 46 μm × 37 μm surface area was made to analyze the quality of transferred monolayer graphene in the polished side of the D-shaped fiber. A linewidth of the order of 30 cm^−1^, full-width at half-maximun (FWHM), corresponds a monolayer while around 40 cm^−1^ indicates bilayer graphene^25^. It can be observed monolayer graphene (green color) was present throughout the polished part of the analyzed area with some bilayer regions (yellow color), as it is usual observed in CVD graphene. All samples used in this experiment showed a surface fully covered mostly by monolayer graphene.

### Polarization relative extinction ratio analysis

#### Measurements

**Figure 2:**
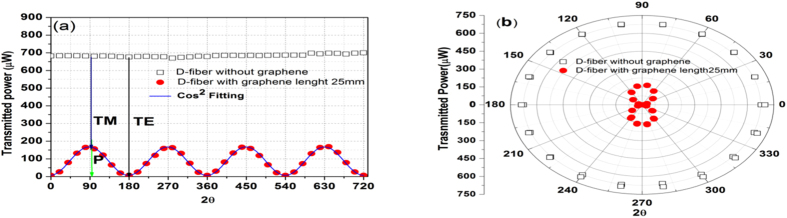
Polarization measures. **(a)** Transmitted power in a D-shaped fiber sample as a function of the angle of the incident linear polarization for sample with and without graphene. The sample with graphene has a core-graphene distance of 1 μm and length of 25 mm. It shows the transverse electric (TE) and transverse magnetic (TM) polarization dependent transmission. **(b)** The same transmitted power in polar coordinates.

The polarization dependent loss of the monolayer graphene D-shaped fiber sample was performed by rotating input power of 754 μW with linear polarization from 0 to 360° degrees. The result is shown in the Fig. 2(a) (black open squares), where no polarization dependent absorption was observed. Thereafter, the sample was characterized after the graphene had been transferred, the results is shown in Fig. 2(a) (red filled circles). A squared cosine function was fitted for the experimental data (blue line curve). The results in Fig. 2(a) were measured in a sample with core-graphene distance of 1 μm and graphene length of 25 mm (identified in the legend as 25-1), the same measurementes were made for all samples. From these curves, we observed the polarization dependent loss due to the graphene varying according to transverse electric (TE) or transverse magnetic (TM) polarization relative to the graphene’s plane^26^. The orthogonal modes are identified in Fig. 2(a), as well the polarization relative extinction ratio (P) which depends of the difference between them. The polarization relative extinction rate was calculated by P = 100(1 - P_TE_/P_TM_), where P_TE_/P_TM_ is the ratio between the power of the modes TE and TM relative to the graphene’s plane. Without graphene, the insertion loss of the D-shaped optical fiber was 0.60 dB, whereas with graphene the TE and TM losses for this sample were 20 and 6.1 dB respectively, resulting in an extinction ratio of 96%. The Fig. 2(b) shows the same plotting in polar coordinates where we observe the graphene-light interaction behavior for polarization angles.

Table 1 presents the extinction ratio measurements of two sets of graphene samples. One with core-graphene distance (h = 0), and the graphene length transferred over the surface of the D-shaped fiber (l = 10 to 24 mm); and the other with core-graphene distance h = 1 μm, and graphene length varying from l = 13 to 25 mm. For all samples we measured the fiber loss without graphene, and after that the TM and TE attenuation with graphene. The attenuation in modes TE and TM varies according to core-graphene distance and graphene length, and also graphene quality, in fact the graphene CVD had different defects associated with the growth process such as the inhomogeneity and discontinuity. These defects generate scattering process and high loss in the transmission. There are another factors associated with the loss, the polymethyl-methacrylate (PMMA) thickness and the transfer process, as we will see in the section of simulation. As expected, the attenuation of the TE mode is higher than TM mode and increases with graphene length. However, we did not observe big differences in the attenuation as a function of the core-graphene distance. The extinction ratio also increases with graphene length and we did not observe correlation with core-graphene distance because there are other parameters cited above affecting it. For our purpose, the extinction ratio is the important parameter regardless the graphene length or core-graphene distance.

### Simulation results D-shaped optical fiber with graphene for distances 0 μm and 1 μm and different PMMA thickness

As it can see in Table 1, the influence of distance h and PMMA thickness over the polarization relative extinction ratio was not possible to be detected experimentally. This is due to the manufacturer polishing accuracy of ±0.5 *μm* and PMMA thickness variation between 300 and 400 nm. An approaching to analyze this influence and give a support to the relative extinction ratio polarization measurements, were done by simulation, using the COMSOL Multiphysics software,version 5.1. The attenuation calculated by simulation for each orthogonal mode in each sample is summarized in Table 2 for a PMMA layer of 300 nm thickness and in Table 3 for a PMMA layer with 400 nm. The simulated results present a smaller attenuation compared to the experiments which indicate additional losses caused by imperfections in the graphene and the presence of scattering regions due to the CVD graphene roughness and graphene’s folding^26^. Additionally, the transference process may be affected by the fiber orientation and graphene accommodation over the fiber’s polished surface. Nevertheless, good agreement with the experiments was obtained by analyzing the polarization extinction ratio, since the effects of the polarization independent losses which were not considered in the simulation are excluded with this criterion. According to the simulation the PMMA layer thickness is a determining parameter to the extinction ratio value, this occurs due to the PMMA high refractive index n = 1.49 which shifts the optical mode towards the graphene increasing the light-graphene interaction. Fluctuations in the spin coated PMMA layer may explain some apparent discrepancies in the experimental results, for instance the highest extinction ratio obtained in the sample with core-graphene distance of 1 μm and 13 mm graphene length. Considering the polarization dependent losses, the analysis through the extinction ratio proved to be adequate to eliminate additional losses in the evaluation of the samples quality. A high interaction with the TE radiation in graphene leads to nonlinear effects in the saturable absorption due to the Pauli blocking effect, which was then evaluated in the EDFL.

### Ultrashort pulse generation

**Figure 3:**
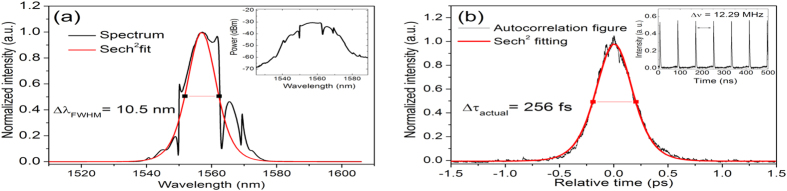
Mode-locking results. **(a)** Output linear spectrum (inset – log scale spectrum) and **(b)** autocorrelation trace (inset – cavity fundamental repetition rate).

The EDFL continuous wave (CW) lasing threshold without the saturable absorber was achieved at a pump power of 15.7 mW. Incorporating the monolayer graphene saturable absorber on the D-shaped fiber, the lasing threshold increased to 20 mW. Non-self-starting mode-locking regime was obtained from 50 to 110 mW pump power, generating soliton-like pulses highly dependent of polarization state. In fact, because of symmetry breaking in the fundamental propagation mode caused by D-shaped optical fiber/graphene configuration and the unequal property of graphene as polarizer and saturable absorber, the mode-locking was activated for high absorption of the TE mode and different polarization states can generate distinct performances in the laser. Figure 3(a) shows the results for the sample with graphene length of 25 mm and core/surface distance of 1 μm, which resulted in the shortest pulses. With this sample the laser generated a soliton spectrum centered at 1557 nm and bandwidth of 10.47 nm for a pump power of 50.7 mW. This spectrum has sidebands associated with soliton-like pulses and dip-type sidebands as a result of the parametric four-wave-mixing effect caused by the strong periodic soliton pulse power variation inside the cavity^27^. This suggests high nonlinearity of the graphene with respect of polarization states inside the cavity, however we believe that the nonlinear polarization rotation (NPR) contribution for ultrashort pulses generation is small. The detailed discussion can be found in the Supplementary Information along with the respective measures in Figures S1 and S2. In Figure 3(a), the inset shows the spectrum in logarithmic and linear scale, respectively. Assuming sech^2^ pulse profile, the measured autocorrelation trace corresponds to 256 fs pulse duration, as shown in Fig. 3(b). The mode-locking pulse train corresponded to the cavity fundamental repetition rate of 12.29 MHz (81.4 ns), exhibited in the Fig. 3(b) inset. The measured output average power was 0.5 mW and the intracavity peak power was calculated to be 1.04 kW. The time-bandwidth product (TBP) of this pulse was 0.331, which is near of the transform-limited value of 0.315 for soliton-like pulses. It is worth mentioning that this result represents the shortest pulse reported in the literature for a single layer graphene used as saturable absorber in an EDFL.

In Table 4, we present the mode-locking result for all samples tested in the EDFL setup. The performance of the graphene as saturable absorber for each sample is related to the pulse duration. The variation in the pump power and peak power in each sample is associated with the nonlinear absorption of the TE mode, graphene length and thickness PMMA.

### Polarization relative extinction ratio and mode-locked pulse duration relation

**Figure 4:**
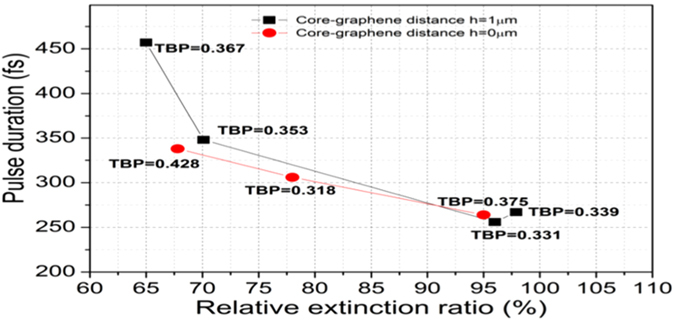
Relative extinction ratio vs pulse duration. Output pulse duration of the mode locked EDFL as a function of polarization relative extinction ratio and time bandwidth product (TBP). Samples with core-graphene distance of 0 μm are represented by red curve, and sample with core-graphene distance of 1 μm are represented by the black-square curve.

The mode-locking performance can be directly related to the sample’s polarization relative extinction ratio as shown in Fig. 4. The shorter pulses are obtained using samples with higher extinction ratios. The samples with extinction ratios higher than 85% generated pulses with duration shorter than 300 fs. This criterion can be used to evaluate the sample quality in any transferred graphene based device, since it excludes losses which may be attributed to imperfections in the graphene or in the transference process. Due to the polarization dependent absorption in the graphene a high extinction ratio means that losses are occurring mainly due to the absorption of the light by the graphene which results in a more efficient saturable absorption in the TE radiation and a lower linear loss insertion in the laser cavity, resulting in a better mode-locking performance.

## Discussion

In this work, we present a simple and high performance criterion for D-shaped optical fiber based on monolayer graphene saturable absorber for the generation of ultrashort pulses in EDFL. With this method, it is possible to associate the polarization relative extinction ratio between the TE and TM radiation with the passively mode-locking optimization. In the EDFL, the samples with polarization relative extinction ratio higher than 85% resulted in pulse duration shorter than 300 fs. The shortest pulse duration of 256 fs was obtained at higher polarization relative extinction ratio of 96%, which is the shortest pulse reported in the literature using CVD monolayer graphene. This criterion can be used to evaluate the quality and performance of several optical devices based on transferred graphene in optical waveguides.

The polarization relative extinction ratio is an important parameter because it is associated with the saturable absorption mechanism which generate ultrashort pulses. The saturable absorption mechanism is associated with electronic transitions from the valence band to the conduction band due to the interaction of light with graphene at high intensities. Due these transitions, the conduction band levels become fully occupied (Pauli blocking), so the graphene becomes transparent for high intensities. In the experiment and simulation results, high relative polarization extinction ratio implies a high absorption at TE mode, responsible for both saturable absorption in graphene, and ultrashort pulses generation.

## Methods

### Graphene saturable absorber fabrication

**Figure 5:**
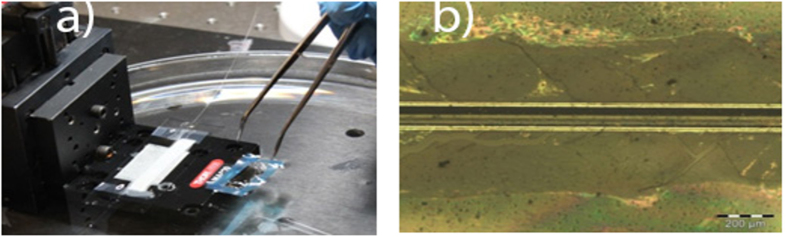
Optical image and photographic. **(a)** Positioning system to the graphene/PMMA transfer process and **(b)** Optical microscopy image of graphene/PMMA film transferred on the side-polished of the D-shaped optical fiber. Take from J. D. Zapata.

The monolayer graphene was produced by chemical vapor deposition (CVD) grown on cooper and the polished D-shaped optical fibers (Phoenix Photonics) samples were provided, with distances from the fiber core to the surface of 0 and 1 μm and polishing lengths of 17, 25 and 30 mm. A total of seven samples were fabricated, three samples with core-surface distance (*h*) of 0 μm with transferred graphene length (*l*) of 10, 18.5 and 24 mm; and four samples with core-surface distance of 1 μm and 13, 14, 19 and 25 mm graphene length. The monolayer graphene was transferred from the cooper to the side-polished of the D-shaped optical fiber by using the wet transfer method^1^. First, a polymethyl-methacrylate (PMMA) polymer film with ~300 nm thickness was spin coated to the graphene on cooper, afterwards the cooper was etched into a 6% persulfate of ammonium (PSA) concentrated solution for 5 hours and then the resulting graphene/PMMA films were washed in a vessel with deionized water. The transference of the graphene/PMMA film from the vessel to the polished side of the fiber was made using the micrometer positioning system shown in the Fig. 5(a).The D-shaped optical fiber was fixed to the positioning basement with the polished face previously oriented upwards; the graphene/PMMA film is surrounded by a blue adhesive tape placed to precision handling. By using tweezers, the graphene/PMMA film was carefully placed on the top of the D-shaped optical fiber, which was carefully lifted using the positioning system, concluding the transference process. The Fig. 5(b) shows the optical microscopy image of the transferred graphene/PMMA film on the polished side of the D-shaped optical fiber, which was taken by optical microscope (Olympus BX51M) using a 5× objective lens.

### Polarization dependent loss experiment

**Figure 6:**
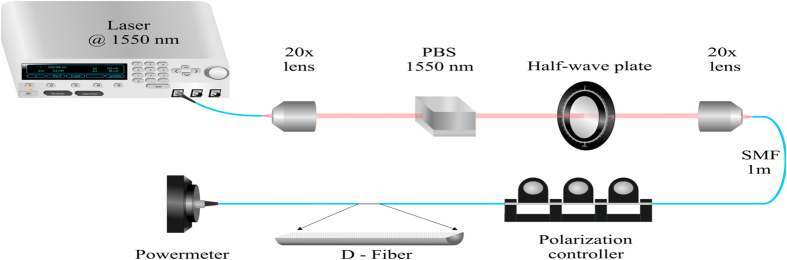
Polarization experimental set up. Experimental setup for measuring the polarization dependent loss in the D-shaped optical fibers with graphene.

The polarization dependent loss of the fabricated samples was characterized with the setup shown in Fig. 6. The laser beam from a laser source Anritsu MG9541A set at 1550 nm was collimated using a 20× objective lens. The beam was then polarized vertically through a polarizing beam splitter (PBS) and the polarization direction was controlled by the half-wave plate. The polarized beam was coupled to a 1 meter length of single mode fiber (SMF) through another 20× objective lens and an in-fiber polarization controller was used to minimize the polarization rotation caused by the SMF. After the polarization controller the SMF was connectorized to the fabricated sample and the output power was measured in a power meter detector.

### Simulation D-shaped optical fiber with graphene for distances 0 μm and 1 μm

**Figure 7:**
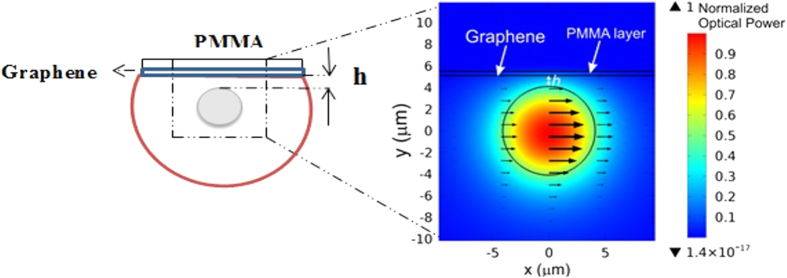
Simulation. Simulated fiber design with the optical power distribution in the TE polarization.

The polarization dependent loss in the fabricated samples was simulated using the COMSOL Multiphysics software,version 5.1. The guided modes were calculated using the finite element method and the attenuation for each polarization was evaluated from the imaginary part of the propagation constant^26^. The simulated transversal section of the D-shaped optical fiber with a single layer graphene is shown in the Fig. 7.

The graphene was modeled using the boundary condition for the tangential magnetic field **H**, **n**×(H_*fiber*_−**H**_*PMMA*_ = σ**E**, where **n** is the unitary vector normal to the graphene’s plane, **E** is the electric field vector and *σ* is the graphene conductivity at the optical frequency^2^ at 1550 nm. Also, it is considering the graphene chemical potential small compared to the photon energy, which means no Pauli blocking effects, the calculated conductivity was σ = (60.8–0.4*i*)*μS*. Figure 7 shows the modal power distribution propagating in the fiber with the arrows representing the electric field direction. In this case the polarization is parallel to the graphene plane (here called TE radiation). The result presented is for a fiber with core-graphene distance *h* = 1 μm; PMMA refractive index 1.49 and 300 nm thickness; fiber core and cladding refractive index 1.4492 and 1.4440 respectively and fiber core diameter 8.2 μm.

### Passively mode-locking experiment

**Figure 8:**
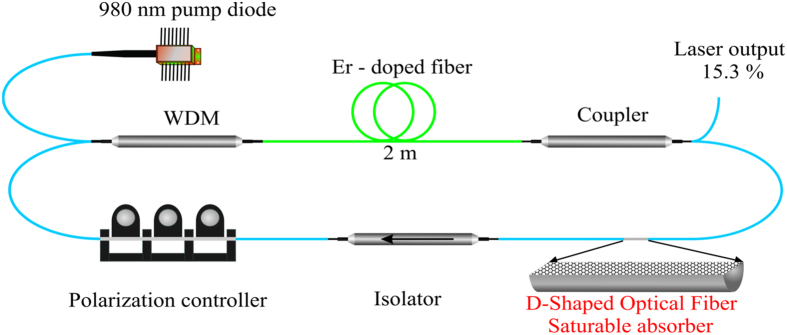
Laser experimental set up. Passively mode locked by graphene saturable absorber on D-shaped optical fiber.

Figure 8 shows the experimental setup of the Erbium-doped fiber laser with total length of 15.4 m. It consists of a 2 m length Erbium-doped fiber with absorption coefficient -33.8 dB/m and dispersion coefficient of −57.0 ps/nm/km at 1550 nm, a 980 nm semiconductor pump laser coupled in co-propagating configuration through a 980/1550 nm WDM, an isolator with 50 dB isolation and 0.07 dB loss at 1550 nm, a polarization controller and an output coupler of 15.3%. The cavity average dispersion was 6 ps/km/nm and the accumulated dispersion was 100 fs/nm. The spectral and temporal mode-locked pulses were evaluated in an optical spectrum analyzer and detected in a 30 GHz photo-detector connected to a 1 GHz sampling oscilloscope. The pulse duration was measured with an autocorrelator and the average power was measured in a power meter.

## Additional Information

**How to cite this article**: Zapata, J. D. *et al.* Efficient graphene saturable absorbers on D-shaped optical fiber for ultrashort pulse generation. *Sci. Rep.* **6**, 20644; doi: 10.1038/srep20644 (2016).

## Supplementary Material

Supplementary Information

## Figures and Tables

**Table 1:** Samples characterization: D-shaped fiber and graphene data, measurements of TM and TE attenuation and extinction ratio calculation.

h: Core-graphene distance	0 μm			1 μm			
l: Graphene length (mm)	10	18.5	24	13	14	19	25
D-fiber loss without graphene (dB)	0.34	0.17	0.50	0.52	0.17	1.20	0.60
TM attenuation with graphene (dB)	4.6	4.7	7.6	6.9	4.6	3.2	6.1
TE attenuation with graphene (dB)	9.5	11.3	20.0	23.8	10.0	7.8	20.0
Relative Extinction Ratio (%)	67	78	95	98	71	65	96

**Table 2:** Simulated results for each fabricated sample (PMMA thickness 300 nm).

h: Core-graphene distance	0 μm			1 μm			
TM attenuation (dB/mm)	0.027			0.014			
TE attenuation (dB/mm)	0.374			0.191			
l: Graphene length (mm)	10	18.5	24	13	14	19	25
Relative Extinction Ratio (%)	55	77	85	41	43	54	64

**Table 3:** Simulated results for each fabricated sample (PMMA thickness 400 nm).

h: Core-graphene distance	0 μm			1 μm			
TM attenuation (dB/mm)	0.025			0.013			
TE attenuation (dB/mm)	0.605			0.302			
l: Graphene length (mm)	10	18.5	24	13	14	19	25
Relative Extinction Ratio (%)	74	91	96	58	61	72	81

**Table 4:** Mode-locking results for all samples in the EDFL.

h: Core-graphene distance	0 μm			1 μm			
l: Graphene length (mm)	10	18.5	24	13	14	19	25
Relative Extinction Ratio (%)	**67**	**78**	**95**	**98**	**71**	**65**	**96**
Δτ: Pulse duration (fs)	**338**	**306**	**264**	**267**	**348**	**457**	**256**
Δλ: Bandwidth (nm)	10.3	8.5	11.5	10.3	6.3	8.6	10.5
λ_c_: Central wavelength (nm)	1562	1565	1557	1560	1563	1564	1557
Time-bandwidth product	0.428	0.318	0.375	0.339	0.353	0.367	0.331
Pump power (mW)	62	63	73	69	43	102	51
Peak power (kW)	1.85	1.81	2.03	1.50	0.50	3.44	1.04
