# Supplementary material for: Efficient graphene saturable absorbers on D-shaped optical fiber for ultrashort pulse generation
Source: Sci Rep. 2016 Feb 9;6:20644. doi: 10.1038/srep20644 (PMC4746624; doi:10.1038/srep20644)
Supplement: Supplementary Information [file srep20644-s1.pdf]

Supplementary Information for

Efficient graphene saturable absorbers on D-shaped optical fiber for ultrashort pulse generation

J. D. Zapata[1,2], D. Steinberg[1], L. A. M. Saito[1], R. E. P. de Oliveira[1], A. M. Cárdenas[2] and E. A. Thoroh de Souza[1,*]

[1]*MackGraphe - Graphene and Nanomaterials Research Center – Mackenzie Presbyterian University - São Paulo/SP – Brasil*

[2] *Universidad de Antioquia - Medellin – Colombia*

Tel.: +55 11 2114-8868; Email: *thoroh@mackenzie.br

Table of contents

Measures of pulse duration and laser bandwidth as function of cavity length

Supplementary Figures S1-2 and Figure Legends

Nonlinear polarization rotation (NPR) effect in the mode-locking performance

We discuss the possible contribution of nonlinear polarization rotation (NPR) in the mode-locking performance by changing the initial cavity length (15 m). The NPR effect depends on the nonlinear phase $\Delta\phi_{NL}$, which depends on fiber length, fiber nonlinearity γ, and peak power; therefore NPR depends on cavity length. For generate ultrashort pulses with NPR technique, it would be necessary a set of polarization-controll+SMF +polarizer acting as a saturable absorber. In this case the laser performance would change significantly for different cavity lengths, as the phase-matching conditions changes.

Supplementary Figure S1 shows the measurements for all cavity lengths given by 12, 13, 15, 17 and 20 m. We observed that by increasing the cavity length from its initial length (15 m) to 17 and 20 m narrows the output spectra and subsequently broadens the pulses, since the initial pulse was near to the Fourier-transform-limit. And by decreasing the cavity length to 13 and 12 m broadens the output spectra and subsequently shortens the output pulses. Supplementary Figure S2 shows the behavior of pulse duration and laser bandwidth as a function of cavity length. The bandwidth (red curve) increases from 6.4 to 15.6 nm as the cavity length decreases from 20 to 12 m. And the pulse duration (black curve) decreases from 1100 fs to 330 fs. The pulses are chirped and can be shortened by dispersion compensation inside or outside the laser cavity. This behavior is consistent with intracavity dispersion management rather than NPR.

Therefore, these results demonstrated that the saturable absorption of the monolayer graphene is the main effect that contributes to the ultrashort pulse generation in these lasers. Nevertheless, the NPR effect may have a small contribution to the mode-locking performance.

In conclusion, the use of D-shaped fiber optimizes the interaction between the pulse and the monolayer graphene allowing a better balance between gain and saturable loss inside the cavity, which can shorten pulses until the limit of graphene response.

<table>
<tr><td>Bandwidth: 15.6 nm (Cavity length = 12 m)</td><td>Pulse duration: 330 fs</td></tr>
<tr><td>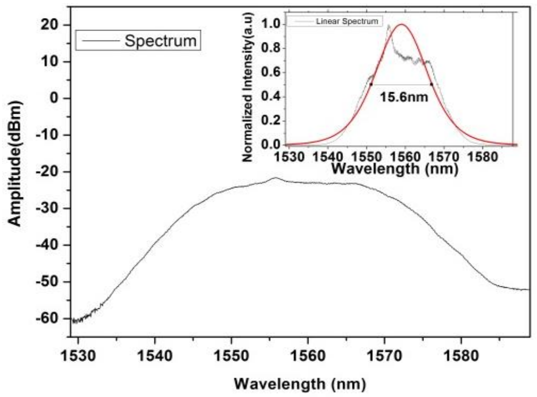
</td><td>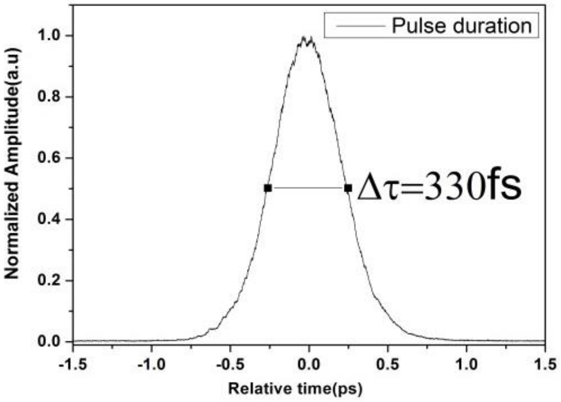
</td></tr>
<tr><td>Bandwidth: 13.4 nm (Cavity length = 13 m)</td><td>Pulse duration: 362 fs</td></tr>
<tr><td>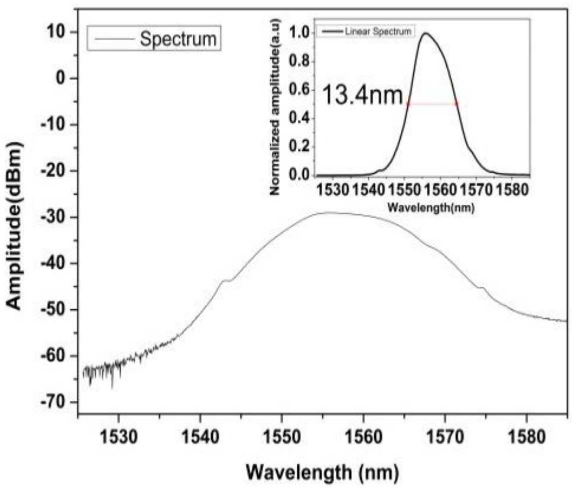
</td><td>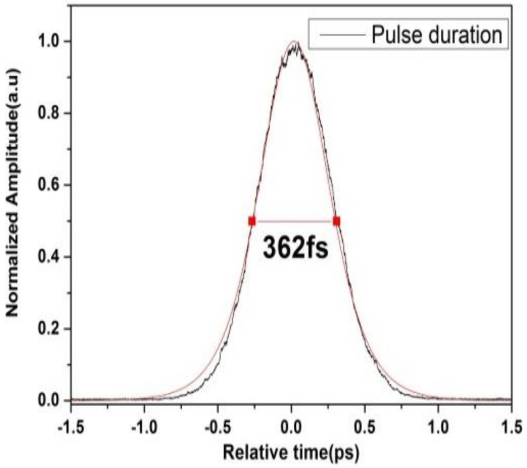
</td></tr>
<tr><td>Bandwidth: 11.8 nm (Cavity length = 15 m)</td><td>Pulse duration: 280 fs</td></tr>
<tr><td>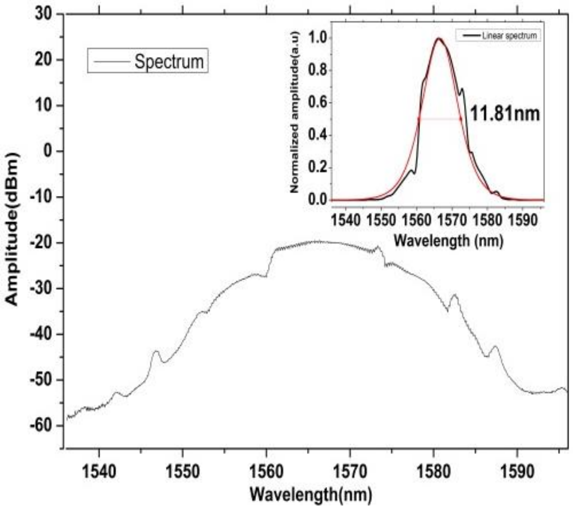
</td><td>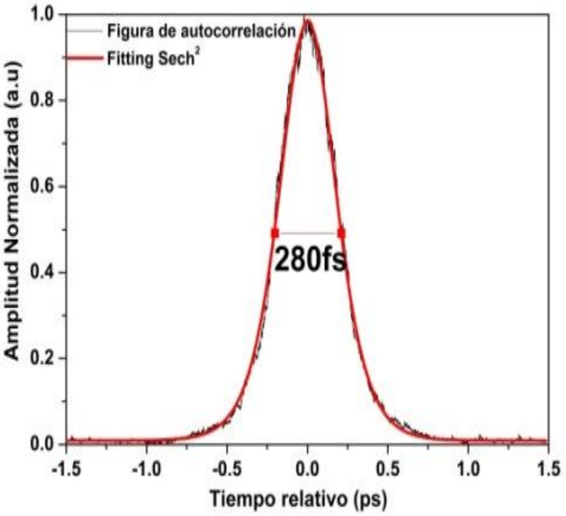
</td></tr>
</table>

Bandwidth: 8.5 nm (Cavity length = 17 m)	Pulse duration: 629 fs
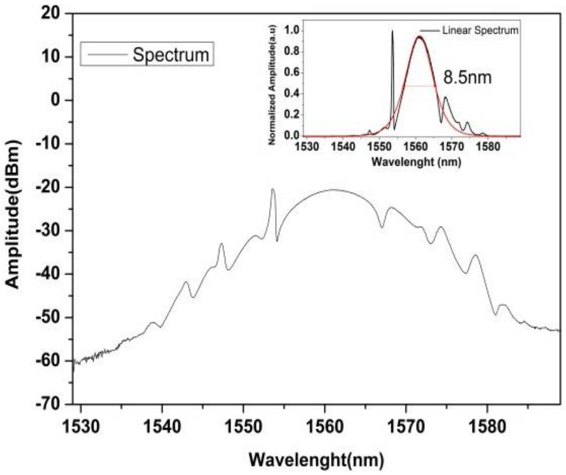	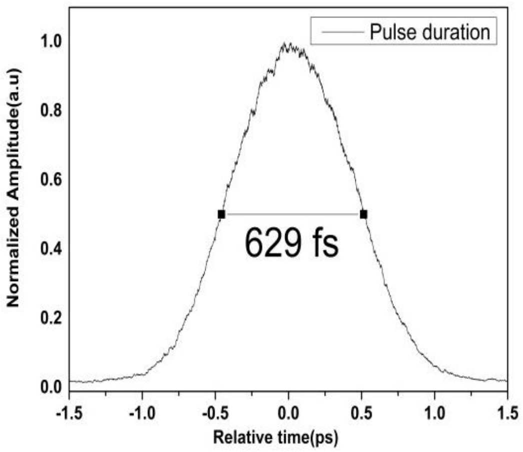
Bandwidth: 6.4 nm (Cavity length = 20 m)	Pulse duration: 1100 fs
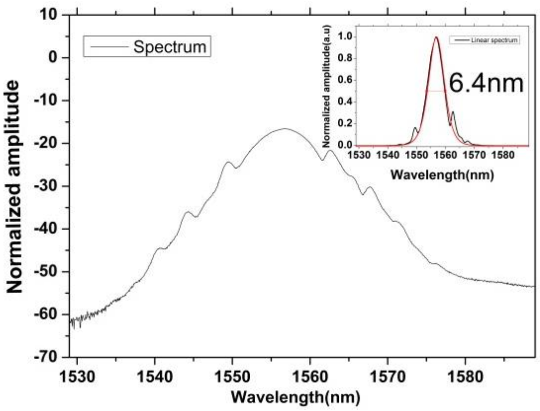	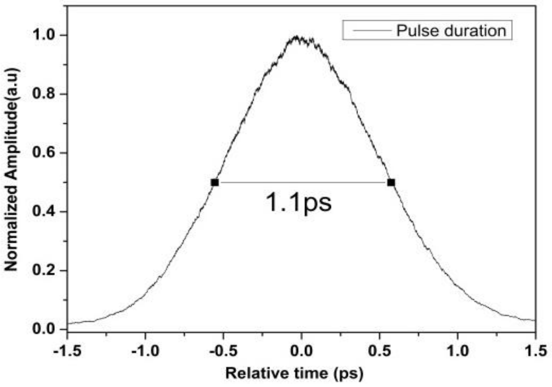

Supplementary Figure S1. Measures of the laser bandwidth and pulse duration for different values of cavity length: 12, 13, 15, 17 and 20 m.

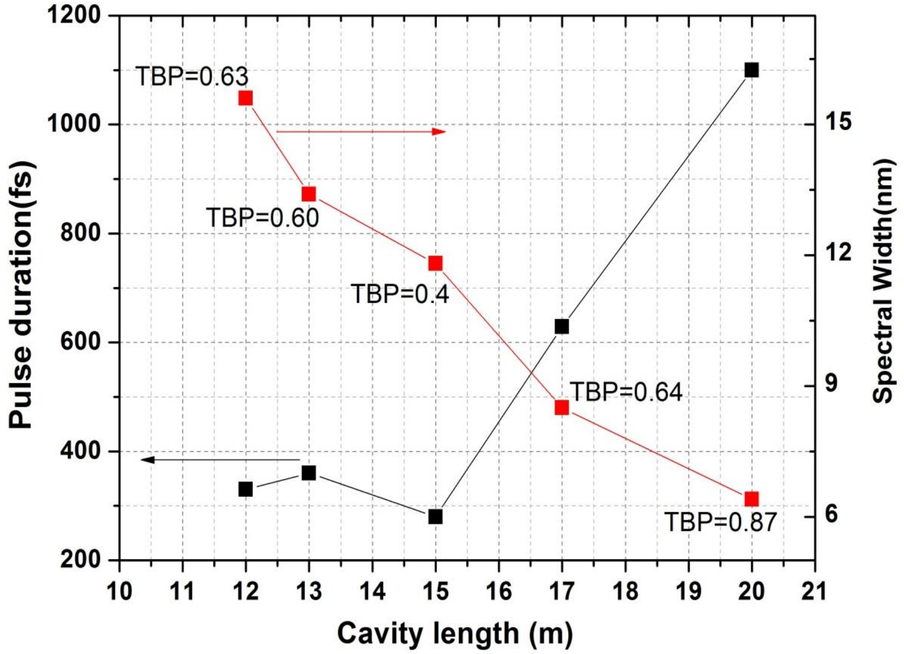

Supplementary Figure S2. Bandwidth and pulse duration as a function of cavity length.
